# Early impact of aortic wrapping on patients undergoing aortic valve replacement with mild to moderate ascending aorta dilatation

**DOI:** 10.1186/1749-8090-5-58

**Published:** 2010-08-06

**Authors:** Keng-Leong Ang, Furqan Raheel, Amrita Bajaj, Andrzej Sosnowski, Manuel Galiñanes

**Affiliations:** 1 Department of Cardiothoracic Surgery, Glenfield Hospital, Groby Road, Leicester, LE3 9QP, UK; 2 Department of Radiology, Glenfield Hospital, Groby Road, Leicester, LE3 9QP, UK; 3 Department of Cardiac Surgery, Area del Cor (ACOR) and Research Institute, University Hospital Vall d'Hebron, Universitat Autònoma de Barcelona, Pg. Vall d'Hebron 119-129, 08035 Barcelona, Spain

## Abstract

**Background:**

The management of mild to moderate dilatation of the ascending aorta of less than 5 cm is controversial, particularly when concomitant surgical correction of aortic valve is required. We investigate the impact of a simple method of aorta reduction using Dacron graft wrapping during aortic valve replacement on the rest of the aorta.

**Methods:**

We studied 14 patients who had ascending aorta dilatation of 4-5 cm before undergoing aortic wrapping during their aortic valve replacement and compared with their post-operative imaging within a month.

**Results:**

The diameters of the ascending aorta wrapped with the Dacron graft were significantly reduced within 4 weeks after surgery from 44.7 ± 2.6 to 33.6 ± 3.9 mm (p < 0.001). This was associated with significant reduction in the diameter of rest of ascending aorta: coronary sinuses (from 37.9 ± 4.9 mm to 33.3 ± 6.1 mm; p < 0.001), sinotubular junction (from 33.2 ± 4.7 mm to 30.6 ± 4.4 mm, p = 0.02), and aortic arch (from 34.7 ± 4.3 mm to 32.6 ± 4.1 mm, p = 0.03).

**Conclusions:**

Reduction of ascending aortic dilatation by wrapping with a Dacron graft in this preliminary study is associated with favourable early reversed aortic remodelling. This supports the hypothesis that correction of mild-moderate dilatation of the ascending aorta with Dacron wrapping at the time of aortic valve surgery may prevent the progression of the dilatation, although the long-term study on a larger population is needed to confirm its benefits.

## Background

While it is generally accepted that ascending aorta dilatation beyond 5 cm should be surgically replaced, opinions on how mild to moderate dilatation of the ascending aorta of less than 5 cm should be treated are divided[1]. In these cases, and in view of the higher surgical risk of ascending aorta replacement, some surgeons favour a "watch and wait" approach, until such a time when surgery is indicated[2]. By contrast, other surgeons recommend early surgical intervention, especially when patients also need surgical correction of concomitant aortic valve pathology, in view of the increased risk of subsequent rupture and dissection[1,3,4]. Different surgical options have been used in such circumstances, ranging from composite graft replacement to more conservative aortic reduction by aortoplasty with or without external reinforcement. However, there is little information on the natural history of the remaining aorta following such procedures.

Here, we report our preliminary experience with a simple method of ascending aorta reduction using Dacron graft wrapping in patients with ascending aortic dilatation of 4.0 - 5.0 cm during aortic valve procedure, and its early favourable impact on the rest of the aortic dimensions following surgery.

## Methods

Between January 2006 to December 2008, patients with documented ascending aortic dilatation of 4.0 - 5.0 cm on CT or MRI scan as part of their pre-operative cardiac surgery workup were identified. In these patients, the reduction of ascending aorta was carried out as described below as an additional procedure to other cardiac operations. For inclusion in the study, patients must have CT scan within 4 weeks after surgery during their routine post-operative follow-up.

Following the initiation of cardiopulmonary bypass, the ascending aorta was freed from its precardial reflection proximally from the sinotubular junction (STJ) and as far up to the origin of the innominate arterial trunk. To perform the aortic valve procedure, a transverse aortotomy was made 1 cm above the STJ, that was directly closed at the end of the aortic valve correction. Just before coming off cardiopulmonary bypass, a Dacron graft of 2.5 - 3.5 cm diameter was cut longitudinally and passed around the ascending aorta to cover its entire length from the STJ to the brachiocephalic artery. Then, the ascending aorta was reduced to the diameter of the Dacron graft by approximating the edges of the Dacron with a continuous 3/0 prolene suture. The aim was to reduce the size of the ascending aorta to a diameter less that 3.5 cm. Therefore, the size and length of Dacron graft was determined by intra-operative measurements of the aorta from the STJ to the origin of the brachiocephalic arterial trunk so that the graft can fix snugly around the aorta at the end of the procedure.

Three patients required coronary artery bypass grafting (CABG) using saphenous vein grafts (SVGs). In one patient, the proximal end of the SVG was attached to the ascending aorta through an opening made in the Dacron graft. The remaining 2 patients had their proximal SVGs attached to ascending aorta distal to the Dacron graft.

The aortic diameter at the STJ, the site of maximal dilatation of the ascending aorta, the coronary sinuses and the aortic arch were measured both at before and after surgery by an experienced observer. The post-operative course was also noted.

Continuous variables that were normally distributed were presented as mean ± standard deviation (SD), and differences between within groups were compared using paired t-tests. Non-normally distributed variables were presented as median with their interquartile range, and analysed using the appropriate non-parametric tests.

The study was approved by the local Ethics Committee and, because this was a retrospective analysis of a procedure previous reported and of investigations performed as part of the standard care, patient's consent was not required.

## Results

During the study period, we identified 14 patients with maximal ascending aortic diameter between 4-5 cm pre-operatively fulfilling the study criteria. There were 11 males and 3 females, and the mean age at surgery was 60 ± 13 years old. Two patients had history of diabetes. Eleven patients had history of hypertension but blood pressure was adequately controlled before and after surgery. Nine patients presented with aortic stenosis, while the remainder predominantly had aortic regurgitation. At surgery, the aortic valve was tricuspid in 7 patients, bicuspid in 4 patients and pseudo-bicuspid in the remaining patients. All patients had tissue aortic valve replacement (AVR) plus reduction of ascending aorta with Dacron graft. In addition, 3 of the patients had CABG with SVG as described above. The aortic wrapping, that was performed at the end of the other surgical corrections had no effect on aortic cross-clamp time and only resulted in small lengthening of the overall CPB. One patient had to be re-opened due to haemodynamic instability and left ventricular dysfunction already present before surgery. The median (interquartile range) postoperative stay was 8 (7) days. However, there was no early morbidity or mortality directly associated with the aortic wrapping.

**Figure 1:**
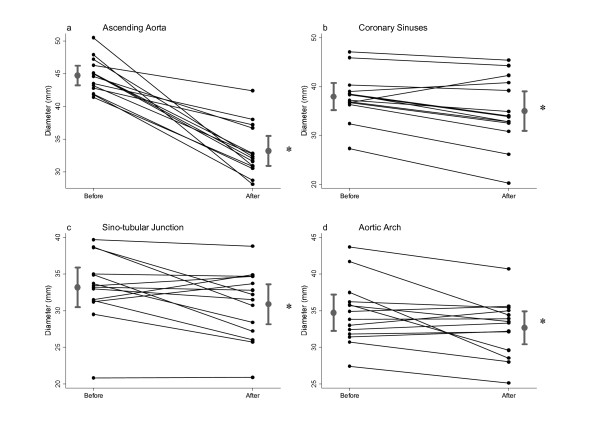
**Mean values of the dimensions of the ascending aorta (a), coronary sinuses (b), sinotubular junction (c) and aortic arch (d) before and after wrapping of the ascending aorta with a Dacron graft**. *p < 0.05 versus before correction values.

Figure 1(a-d) shows that, as expected, the diameters of the ascending aorta wrapped with the Dacron graft were significantly reduced within the first 4 weeks after surgery from 44.7 ± 2.6 to 33.6 ± 3.9 mm (p < 0.001). Interestingly the diameters of the coronary sinuses were also significantly reduced from 37.9 ± 4.9 mm before surgery to 33.3 ± 6.1 mm after the aortic wrapping (p < 0.001). A lower but still statistically significant reduction of the STJ size from 33.2 ± 4.7 mm to 30.6 ± 4.4 mm was also seen (p = 0.02). The changes in the dimensions of the aorta distal to the wrapping were associated with modest but still significant reduction of the maximal diameter of the aortic arch from 34.7 ± 4.3 mm before surgery to 32.6 ± 4.1 mm after aortic wrapping (p = 0.03). It is worth noting that the observed remodelling of the aorta proximal and distal to the wrapping was unrelated to the aortic valve pathology.

## Discussion

Mild to moderately dilated ascending aorta is encountered in 5-15% of patients needing AVRs[4]. However, its management is controversial. There is very little information on the natural history of dilated ascending aorta after AVR. It is debatable whether the correction of aortic valve pathology alone is adequate to stop the progression of the dilatation of the aorta, especially when the native aortic valve is bicuspid[5]. Furthermore, there is an increased incidence of rupture and dissection in these patients after AVR alone[6]. Thus, the current recommendations favour early definitive surgical intervention in these patients if they require open heart procedure[1]. However, the determination of the type of treatment remains unclear and a conservative surgical approach with reduction aortoplasty, with or without external support, or with external support alone have been advocated for these circumstances as they can be performed without increasing the operative risk, [3,4,7,8] but the effect of such treatments on the remaining aorta remains unknown.

Here we are reporting the preliminary findings of the use of a simple method of reducing the mild to moderate dilatation of the ascending aorta using wrapping of the dilated segment with a Dacron graft tube when associated aortic valve surgery. This procedure does not require the opening of the aorta neither the extension of the aortic cross-clamp time. It can be performed relatively quickly within 5-10 minutes, and is safe, with no significant morbidity or mortality. The procedure was performed in combination with other cardiac surgical corrections but theoretically it could also be used safely in isolation.

The most important finding of this study is the reduction of the diameter and reversed remodelling of the aorta not only at the site of dilatation but also proximally and distally to the aortic wrapping, a benefit seen early after surgery. Despite the small number of patients, the reduction in diameter at different aortic sites was statistically significant, suggesting that correction of dimensions of a dilated ascending aorta at an early stage, and before irreversible anatomical changes take place, results in the rapid reversed remodelling of the rest of the aorta, probably due to the restoration of normal blood flow haemodynamics. This reversibility could support the case for an early intervention in mild to moderate ascending aortic dilatation so that, in addition to stopping its progression, the future abnormal dilatation in the remaining aorta can be prevented.

Although we did not observed any early complications, there was the concern in previous studies of reduction aortoplasty re-enforced with Dacron wrapping that Dacron grafting could potential erode into the aorta[9]. However, in a recent study, no aortic erosion was noted on histology in the autopsy of a patient whom died from other causes[10]. Nevertheless, like any foreign material, there is always a risk of infection, and therefore the same precautions as with valve surgery should be taken following the procedures.

This pilot study is limited by the lack of a control group, and the fact the measurements were made by a single, but experienced observer in aortic anatomy. The findings here also related to early post-operative outcomes, hence a long-term follow-up, with a bigger population in a prospective randomized study is necessary to ascertain the full benefits of this procedure as well as its safety.

## Conclusions

This preliminary study shows that surgical wrapping of mild to moderate ascending aorta dilatation at aortic valve surgery is associated with early reversed remodelling of the remaining proximal and distal aorta, without any short-term complications. Our findings support the hypothesis that correction of mild-moderate dilatation of the ascending aorta with Dacron wrapping at the time of aortic valve surgery may prevent the progression of the dilatation, although the long-term study on a larger population is needed to confirm its benefits.

## Competing interests

The authors declare that they have no competing interests.

## Authors' contributions

All authors fulfilled the stated authorship requirements as set out in the author's instruction. KA & FR contributed equally both as first authors, and were involved in all stages of the study and manuscript preparation. AB was mainly involved in the analysis of aortic measurements. AS & MG conceived the idea and participated in its design and coordination and helped to draft the manuscript. All authors read and approved the final manuscript.
